# Present and Future Therapeutic Approaches to Barrier Dysfunction

**DOI:** 10.3389/fnut.2021.718093

**Published:** 2021-10-28

**Authors:** Marina Fortea, Mercé Albert-Bayo, Mar Abril-Gil, John-Peter Ganda Mall, Xavier Serra-Ruiz, Alejandro Henao-Paez, Elba Expósito, Ana María González-Castro, Danila Guagnozzi, Beatriz Lobo, Carmen Alonso-Cotoner, Javier Santos

**Affiliations:** ^1^Laboratory for Enteric NeuroScience, Translational Research Center for GastroIntestinal Disorders, University of Leuven, Leuven, Belgium; ^2^Laboratory of Neuro-Immuno-Gastroenterology, Digestive System Research Unit, Vall d'Hebron Institut de Recerca (VHIR), Vall d'Hebron Hospital Universitari, Barcelona, Spain; ^3^Department of Biomedical and Clinical Sciences, Linköping University, Linköping, Sweden; ^4^Department of Gastroenterology, Vall d'Hebron Hospital Universitari, Barcelona, Spain; ^5^Facultad de Medicina, Universitat Autònoma de Barcelona, Bellaterra, Spain; ^6^Centro de Investigación Biomédica en Red de Enfermedades Hepáticas y Digestivas (CIBERHED), Instituto de Salud Carlos III, Madrid, Spain

**Keywords:** epithelial barrier function, intestinal permeability, nutrients, short chain fatty acids, prebiotics, probiotics, mast cell stabilizers, mucoprotectants

## Abstract

There is converging and increasing evidence, but also uncertainty, for the role of abnormal intestinal epithelial barrier function in the origin and development of a growing number of human gastrointestinal and extraintestinal inflammatory disorders, and their related complaints. Despite a vast literature addressing factors and mechanisms underlying changes in intestinal permeability in humans, and its connection to the appearance and severity of clinical symptoms, the ultimate link remains to be established in many cases. Accordingly, there are no directives or clinical guidelines related to the therapeutic management of intestinal permeability disorders that allow health professionals involved in the management of these patients to carry out a consensus treatment based on clinical evidence. Instead, there are multiple pseudoscientific approaches and commercial propaganda scattered on the internet that confuse those affected and health professionals and that often lack scientific rigor. Therefore, in this review we aim to shed light on the different therapeutic options, which include, among others, dietary management, nutraceuticals and medical devices, microbiota and drugs, and epigenetic and exosomes-manipulation, through an objective evaluation of the scientific publications in this field. Advances in the knowledge and management of intestinal permeability will sure enable better options of dealing with this group of common disorders to enhance quality of life of those affected.

## Introduction

This manuscript belongs to a series of articles dealing with the role of intestinal barrier dysfunction in the origin of chronic inflammatory disorders. Previous papers in this monography review the anatomical, molecular, microbiological, immunological, and pathophysiological bases that link intestinal permeability to the development of chronic conditions within the gastrointestinal tract. Some studies also point to a prominent role of abnormal responses to food and microbial antigens, and toxins, resulting from the alteration of the intestinal epithelial permeability, in the generation of symptoms and signs common to functional diseases of the digestive tract. Although the theoretical basis for this hypothesis is apparently solid, it is nonetheless true that translation from pathophysiological alterations to clinical manifestations relies mostly on *in vitro* and *ex vivo* studies and preclinical models. Therefore, more evidence from clinical trials is needed to determine their role in the management of these diseases.

Despite outstanding advances on the pathophysiology and molecular mechanisms underlying barrier abnormalities, currently we have no universal standards to accurately determine the magnitude of the problem (see other papers in this monography). In this sense, functional barrier parameters such as lactulose/mannitol ratio and maybe certain blood markers may be more indicative for intestinal barrier function than secondary parameters such as levels of tight junction protein expression. This lack of standardization generates confusion impeding further actions of regulatory agencies and many health-care professionals doubt the validity, usefulness and clinical applicability of the different techniques used for the determination of intestinal permeability.

Closely related to the scant clinical evidence linking permeability alterations with inflammatory disorders of the digestive system and other body systems is the insufficient development of molecules or drugs aimed at controlling this function. This is despite the large number of potential therapeutic targets in which a regulatory role has been evidenced in both the pore pathway and the leak pathway. In addition, the modulation of the microbiota and its metabolites, through nutrition, can also play an important role in the therapeutic armamentarium of altered intestinal permeability.

**Figure 1:**
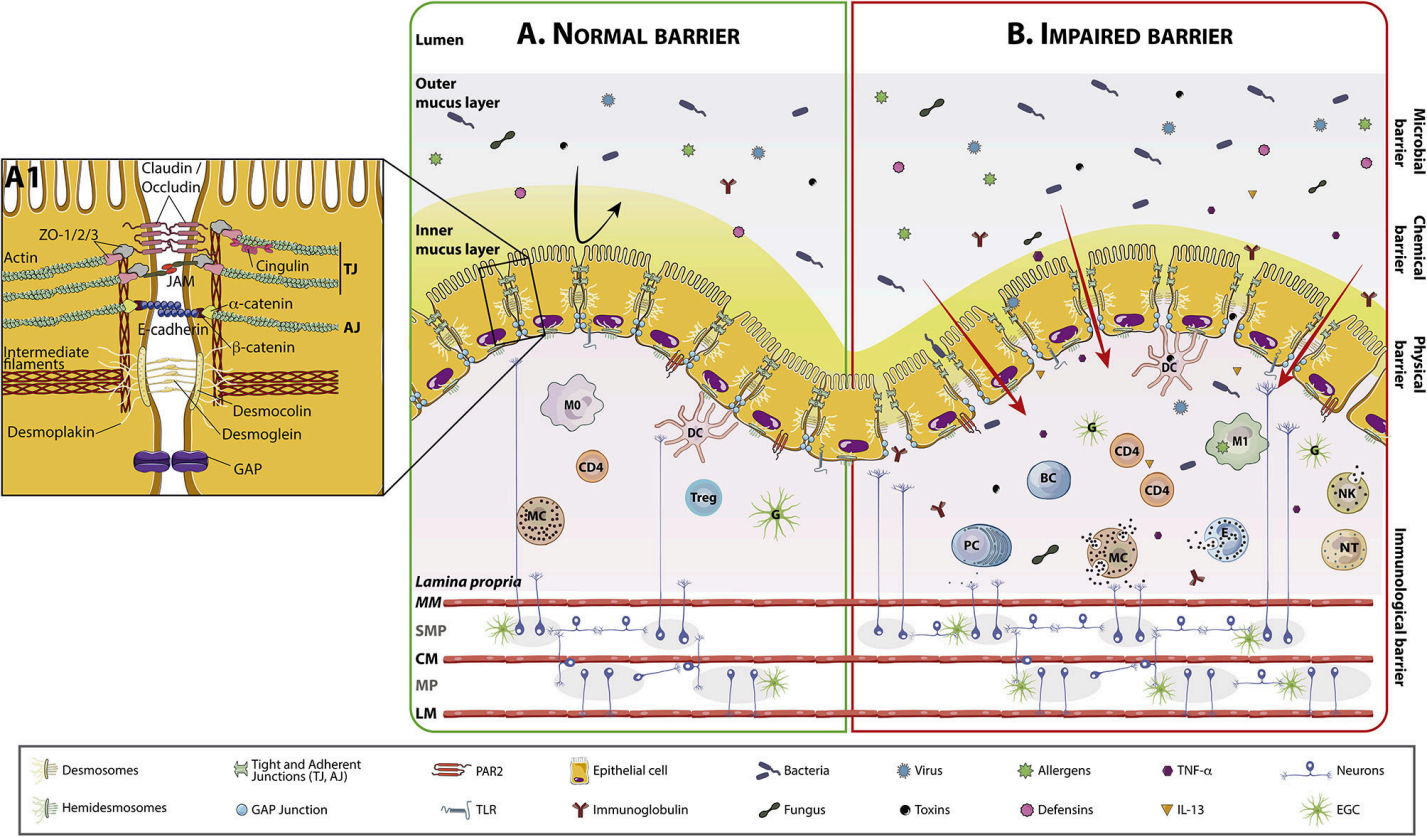
Intestinal barrier anatomy and its components in normal and impaired conditions. The intestinal mucosa comprises a layer of polarized, columnar epithelial cells next to a subepithelial region that contains the *lamina propria*, the enteric nervous system, connective tissue, and muscular layers. On top of columnar cells there is the mucus layer. Normal mucus is 98% water, being the rest glycosilated proteins (mucins) and glycolipids. In the colon, mucus has an outer layer, densely colonized by bacteria, fungus, virus, and able to retain toxins and allergens, and a mostly sterile inner layer where immunoglobulins (mostly secretory-IgA), and defensins such as lysozyme are present. The inner mucus layer is dense and attached an ~50 μm thick and the outer layer is loose and unattached and about 100 μm thick. The small intestine has only one single mucus layer, which is much thinner than the mucus layer in the large intestine. The *lamina propria* includes a diffuse lymphoid tissue constituted by macrophages, dendritic cells, plasma cells, *lamina propria* lymphocytes, MCs, eosinophils and occasionally, neutrophils. **(A)** The intestinal barrier in homeostasis, where cells are closely attached by intercellular junctions (TJs, adherens junctions, desmosomes and GAP junctions) represented in detail in (A1). **(B)** Impaired intestinal barrier, with increased trans and paracellular passage of lumen contents. This increased transport activates the immune system and cell recruitment and degranulation in the *lamina propria*. MC and PC are able to modulate the ENS interacting with SMP/MP neurons and with EGCs. (A1) Representation of intercellular junctions. Intercellular junctions are primary responsible for nutrient absorption and water and chloride secretion. Intercellular junctional complexes, including TJs, adherens junctions, gap junctions, and desmosomes, are dynamic structures that restrict the passage of molecules: 4–5 Å at the villus tip to over 20 Å at the base of the crypt in the small bowel. The integrity and structure of epithelial cells are mostly modulated by the cytoskeleton, mainly by actin, myosin, and intermediate filaments. Cells adhere to the basement membrane through hemidesmosomes. TJs are primarily made up of CLDNs, OCLNs, and JAM proteins, which are connected through *zonula occludens* and cingulin to the cytoskeleton. Adherens junctions include cadherins such as E-cadherin, which binds catenins (α and β) connected to the cytoskeleton. Desmosomes are mainly comprised of desmocollin and desmoglein, which interact with desmoplakin, in turn connected to the intermediate filaments. AJ, Adherens junctions; BC, B Cell; CD4, Lymphocyte T helper CD4^+^; CLDN, Claudin; CM, Circular muscle; D, Desmosomes; DC, Dendritic cell; EGC, Enteric glial cell; ENS, Enteric nervous system; IL-13, Interleukin 13; JAM, Junctional adhesion molecule; LM, Longitudinal muscle; M0, Macrophages type 0; M1, Macrophages type 1; MC, Mast cell; MM, Muscularis mucosae; MP, Myenteric plexus; NK, Natural killer; NT, Neutrophil; OCLN, occludin; PC, Plasma cell; SMP, Submucous plexus; TJ, Tight junctions; TNF-α, Tumor necrosis factor alpha; Treg, T regulatory lymphocyte.

There are hundreds of publications that have investigated a huge number of molecules involved in intestinal barrier homeostasis, though many data are derived from *in vitro* or animal studies what may not well-represent the physiologic situation in the human organism and may not correctly mimic human pathology. In this article, we will review the evidence related to the use of those molecules or products that offer greater potential for the clinical management of diseases that have been more consistently associated with intestinal epithelial barrier (IEB) dysfunction. We will focus our review on the paracellular route as the main target of the epithelial barrier breakdown and as an early event whose loss of functional integrity likely facilitates transepithelial antigen penetration, and the stimulation of immunological responses, further increasing paracellular epithelial permeability and promoting the development of low-grade mucosal inflammation (Figure 1) (1).

The potential market for intestinal permeability regulatory products is unknown but intuitively ample. However, it remains to be established the mechanistic link between alterations in intestinal permeability and specific diseases to estimate how many patients could benefit from better therapies for intestinal permeability and the direct and indirect cost derived from attending these people.

Finally, this manuscript is not intended as a systematic review of the literature concerning intestinal permeability and its management. We just want to raise awareness on the potential of targeting intestinal permeability to improve gut mucosal inflammation and related clinical manifestations. However, we also want to make clear that improving barrier integrity does not mean that inflammation and immune activation are interrupted because this deserves further evidence and possibly complementary approaches to manage microbiome and immune system defects.

## Approach to Management

### Nutrients

**Figure 2:**
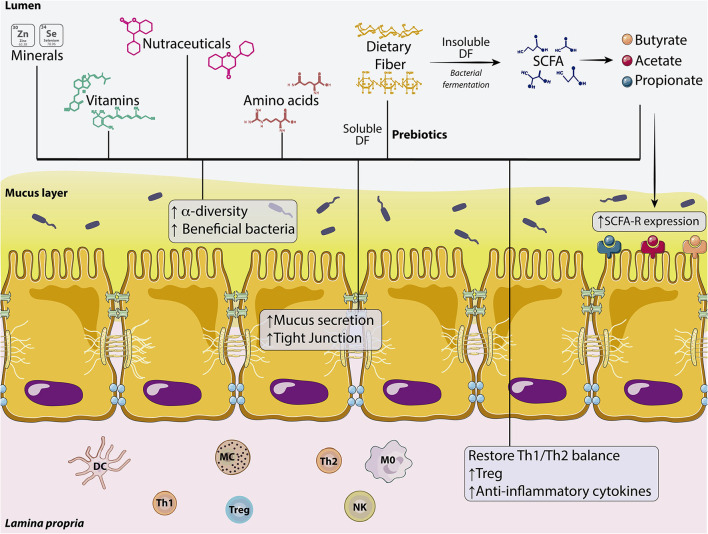
Dietary components involved in the regulation of intestinal permeability. Effects of minerals, vit, nutraceuticals, amino acids, soluble DFs (prebiotics), and SCFAs on the intestinal barrier. These dietary components are capable of affecting the microbiota by increasing α-diversity and the number of beneficial bacteria. DFs also enhance mucus secretion from the IEB and increase the expression of TJs proteins. In the *lamina propria*, dietary compounds increase T reg population, but also the production of anti-inflammatory cytokines restoring the Th1/Th2 balance. Fermentation of insoluble DF to SCFA (butyrate, acetate, and propionate) also increases the expression of SCFA receptors. DC, Dendritic cell; DF, Dietary fiber; IEB, Intestinal epithelial barrier; M0, Macrophage type 0; MC, Mast cell; NK, Natural Killer; Th1, T helper lymphocyte type 1; Th2, T helper lymphocyte type 2; Treg, T regulatory lymphocyte; SCFA, Short chain fatty acid; Vit, Vitamins.

Nutrition has a key role in shaping gut microbiota (2) whereas processing of food by gut microbiota releases byproducts and metabolites that influence the functioning of the intestinal barrier and mucus layer integrity (2, 3) in health and disease (4, 5) (Figure 2).

#### Dietary Fibers, Prebiotics, and Short-Chain Fatty Acids

The international CODEX Alimentarius Commission defined in 2009 dietary fibers (DF) as “carbohydrate polymers with 10 or more monomeric units which are not hydrolyzed by the endogenous enzymes in the small intestine of humans” (6). In terms of solubility, DF differ in their chemical properties (7, 8). Insoluble fibers mainly contribute to stool bulk whereas soluble fibers are metabolized by the host microbiota, also contributing to maintain eubiosis (9). In fact, many of these fibers can be considered as prebiotics because they are resistant to the acidic pH of the stomach, not hydrolyzed by mammalian enzymes, not absorbed in the GI tract, but fermented by intestinal microbiota, and selectively stimulate the growth and/or activity of the intestinal microbiota, particularly *Bifidobacteria* and *Lactobacilli* (10, 11), to improve host's health (12). This may be relevant as bacterial dysbiosis is highly associated with intestinal barrier dysfunction and related pathologies such as Inflammatory Bowel Disease (IBD) (13) (see Table 1). In addition, the outer mucus layer is degraded to glycans by the glycan-consuming microbiota and glycans reused by bacteria in the absence of sufficient DF, as shown in a gnotobiotic mouse model, what may lead to erosion of the colonic mucus barrier, promoting greater epithelial access, and lethal colitis by mucosal pathogens (38).

**Table 1:** Dietary fiber role in the recovery of impaired barrier function.

**Dietary fiber**	**Sample**	**Effect/Implicated mechanisms**	**References**
***In vitro***			
β-galactomannan Mannanoligosaccharide Monosaccharide D-Mannose	*Salmonella enterica*-infected intestinal porcine cells IPI-2I (ECACC 93100622)	•Reduction secretion of pro-inflammatory cytokine Salmonella-induced	(14)
β-galactomannan	*S. enterica*-infected Caco-2 cells	•Prevention epithelial barrier function induced disruption induced by the pathogen	(15)
***In vivo***			
5% *Plantago ovata* seeds	HLA-B27 transgenic rats	•Ameliorate development of colonic inflammation and pro-inflammatory mediators •Improve the intestinal cytoarchitecture •Increase butyrate/propionate in intestinal content	(16)
High-amylose corn starch	TNBS-induced colitis male Wistar rats	•Protection against colonic injuries. •Improvement in the SCFA production. •Reduced colonic permeability.	(17)
Dietary pectin	IBD IL10^−/+^ mice	•Reduced inflammatory response in colon •Modulation of pro-inflammatory cytokines and Ig.	(18)
Soluble fiber and corn starch	IBD IL10^−/+^ mice	•Amelioration of clinical disease and inflammatory ileal and colonic lesions development. •Suppression of gut inflammation by Treg cells, IFN-γ, and colonic PPARγ expression	(19)
Dietary cellulose	DSS-induced colitis neonatal C57BL/6J male mice	•Induction of colonic microbiome shifts. •Improvement of intestinal inflammation.	(20)
Psyllium fiber	DSS-induced colitis ICR and BALB/c mice	•Amelioration or resolution of the colonic damage and inflammation.	(21)
GG and PHGG	Chronic kidney disease male induced ICR mice	•Restoration of colonic barrier. •Up-regulation of TJ. •Increase beneficial microflora composition.	(22)
*Plantago ovata*	Broiler chickens	•Increase body weight and small intestine length. •Reduction amount of *E. coli*.	(23)
Grape peel powder	Acute TNBS-induced colitis adult male Wistar	•Reduction of colitis. •Reestablishment intestinal barrier function.	(24)
Lemon peel powder	DSS-induced colitis male BALB/c mice	•Reduction of the intestinal damage. •Protection of TJ barriers. •Suppression inflammatory reaction.	(25)
GOS	Barrier damage LPS-induced mice	•Attenuation of the intestinal barrier damage. •Reduction of inflammatory responses in the jejunum and ileum. •Up-regulation of intestinal TJ. •Down-regulation of pro-inflammatory cytokines.	(26)
Psyllium husk	Chronic large-bowel diarrhea induced dogs	•Decrease defecation frequency. •Improvement stool consistency. •Weight gain promotion.	(27)
AOS	Weaned pigs	•Increase TJ expression, cecal and colonic p-AMPKα, bacteria from Bacteroidetes and Firmicutes phylum and fecal SCFA. •Decrease pro-inflammatory cytokines and NF-κB	(28)
Sulfated polysaccharide	BALB/c mice DSS-induced colitis	•Inhibit colon shortening and oedema forming •Down-regulation of TNF-α, IL-6, IL-1β •Up-regulation of CLDN-1, ZO-1, MUC-2 and SCFA receptors	(29)
**Clinical trials**			
*Plantago ovata* seeds	IBD patients (12 m)	•Safe and effective. •Maintenance UC remission.	(30)
FOS	Active CD patients (3w)	•Increase bifidobacteria. •Enhancement of DC IL-10 production. •Increase TLR expression in l*amina propria*.	(31)
FOS	Active CD patients (4w)	•Not show clinical benefit. •No significant differences in fecal concentration of bifidobacteria and *F. Prausnitzii*.	(32)
Wheat bran	CD patients (4w)	•Consumption was feasible.	(33)
		•No adverse effects. •Improvement health-related QoL and GI function.	
Low-FODMAP	IBS-patients (3w)	•Reduction GI symptoms.	(34)
Non-digestible polysaccharides	Elderly population with GI symptoms (biopsies, *ex vivo*)	•Reduction colonic hyperpermeability.	(35)
Controlled-fiber diet	NAFLD patients (6 m)	•Reduction serum biomarker of permeability. •Positive influence in NAFLD-associated parameters.	(36)
Low-FODMAP diet Traditional dietary advice	IBS-D patients (4w)	•Low-FODMAP improved symptoms and QoL.	(37)

*AOS, Alginate oligosaccharide; CD, Celiac disease; DC, Dendritic cell; DSS, Dextran Sulfate Sodium; FODMAP, Fermentable oligosaccharides, disaccharides, monosaccharides, and polyols; FOS, Fructooligosaccharides; GG, Guar gum; GI, Gastrointestinal; GOS, Galactooligosaccharides; IBS, Irritable Bowel Syndrome; IBS-D, Diarrhea predominant Irritable Bowel Syndrome; IBD, Inflammatory Bowel Disease; IFN-γ, Interferon gamma; Ig, Immunoglobulin; IL, interleukin; m, Months; NAFLD, Non-alcoholic fatty liver disease; PHGG, Partially hydrolyzed GG; PPARγ, Peroxisome proliferator-activated receptor gamma; QoL, Quality of life; SCFA, Short-chain fatty acids; TEER, Transepithelial electrical resistance; TJ, Tight junction; TLR, Toll-like receptor; TNBF, Trinitrobenzene sulfonic acid; Treg, Regulatory T lymphocyte; UC, Ulcerative Colitis; w, Weeks*.

These dietary compounds mainly include inulin-type fructans (inulin, oligofructose, and fructooligosaccharides), galactans, galactooligosaccharides (GOS), and other heteropolysaccharides such as chitosan, starch, alginate, pectin, or dextran, among others. These products have been shown to positively impact intestinal barrier function through different mechanisms after their fermentation by non-pathogenic colonic bacteria. Fermentation of DF by gut microbiota releases short-chain fatty acids (SCFAs). SCFAs are carboxylic acids with aliphatic tails of 1–6 carbons, being the most abundant acetate, propionate, and butyrate (39). SCFAs show a wide range of biological functions including anti-inflammatory responses, modulation of colonic contractility and maintenance of both mucosal immune cell activity and integrity of the IEB, among others (see Table 2). SCFAs, specially at low concentrations, increased transepithelial electrical resistance (TEER) in T84 and Caco-2 cells, what immediately enhanced barrier function of the colonic epithelium through cholesterol-rich microdomain in the plasma membrane and decreased inulin permeability (42, 54–56). These effects seem to be mediated by AMP-activated protein kinase (AMPK) activity and the accelerated assembly of tight junction (TJ) proteins (43, 44).

**Table 2:** Short-chain fatty acids role in the recovery of impaired barrier function.

**SCFAs**	**Sample**	**Effect/Implicated mechanisms**	**References**
***In vitro***			
10 or 100 mM mixed SCFA 10 or 100 mM acetic, propionic or butyric acids	*Ex vivo* male Wistar rats Cecum and colon	•Modulation contractile activity.	(40)
Butyrate and propionate	THP-1 cells	•Inhibition of TNF-α	(16)
Butyrate 2 mM [low] Butyrate 8 mM [high]	Caco-2 cells	•[Low] promotion of intestinal barrier function, increase TEER and decrease inulin permeability. •[High] induce apoptosis, decrease cell viability.	(41)
Mixed SCFA: acetate –propionate –butyrate 80:40:20, 40:20:10, and 20:10:5 (mmol/l)	*ex vivo* colon Sprague-Dawley rats	•TEER: increase by physiological SCFA mixture and individual SCFA (dose-dependent). •Paracellular transport: dose-dependent reduced by mixed SCFA, acetate and propionate.	(42)
80 acetate, 40 propionate, and 20 butyrate (mmol/l)	Caco-2 cells	•TEER increase with acetate (40 and 80 mmol/l) and propionate (20 and 40 mmol/l).	
80 acetate, 40 propionate, and 20 butyrate (mmol/l)Formate, lactate and succinate (50 mmol/l)	T84 cells	•Acetate + propionate: increase TEER dose/time-dependent manner. •Butyrate and formate do not change TEER. •Propionate, acetate and butyrate and lactate: TEER higher 30 min after (50 mmol/l). •Succinate reduces TEER.	
Butyrate 2 mM	Caco-2 cells	•Increase AMPK activity. •Accelerated TJ assembly. •Increase TEER.	(43)
Butyrate at 2, 5, or 8 mM SB203580: p38 MAPK inhibitor	Caco-2 cells	•2 mM: does not modify intestinal permeability. •5- and 8-mM increase permeability. •5 mM + SB203580 restore the permeability.	(44)
Butyrate, propionate, and acetate	YAMC and Caco-2 cells	•SCFA triggers Aryl hydrocarbon receptor-responsive genes. •AhR plays an important role in GI health and in the gut inflammation by the induction of Tregs.	(45)
Acetate, propionate or butyrate with or without LPS	•Caco-2 cells	•Reduction NLRP3 inflammasome and autophagy. •Decrease intestinal barrier disruption.	(46)
Main fecal SCFA (acetate, propionate and butyrate)	*ex vivo* C57BL/6J mice	•Microbial SCFA are modulated by the circadian rhythm. SCFA affects colon contractility.	(47)
Propionic acid (PA)	Intestinal epithelium cells (IEC-6)	•Promotion of cell migration. •Inhibition of NLRP3 inflammasome. •Activation and improvement of intestinal barrier function. •Suppression of TLR4/NF-κB pathway.	(48)
SCFA produced by *E. coli*	Cancer cell lines: colon (HT-29), breast (MCF-7) and leukemia (THP-1)	•Lower cytotoxicity activity. •Decrease production of inflammatory cytokines.	(49)
***In vivo***			
Sodium butyrate	Wistar rat IBS model (WAS)	•Dose-dependent inhibition of allodynia and colonic hyperpermeability.	(50)
GG and PHGG	DSS-induced colitis BALB/c mice	•Improved clinical score. •Up-regulation of colonic TJ. •High fecal SCFA	(51)
Mixed SCFA: Sodium acetate (3 mM), Sodium propionate (1 mM), Sodium butyrate (1 mM)	Sprague-Dawley rat IBS model (WAS)	•SCFA alleviated colonic spontaneous motility •Fecal SCFA reduction in WAS •Up-regulate SCFA colonic receptors in WAS.	(52)
Acetate, propionate, and butyrate at 0.5, 1, 5, 10, 30 mM	Neonatal BALB/c mice-IBS model rectal 1% acetic ac.	•Dose-dependent reduction colonic transit rate.	(53)

*AhR, Aryl hydrocarbon receptor; AOS, Alginate oligosaccharide; CLDN, Claudin; DSS, Dextran Sulfate Sodium; GG, Guar gum; IBS, Irritable Bowel Syndrome; IL, Interleukin; LPS, Lipopolysaccharide; MUC, Mucin; NF-κB, Nuclear factor – kappa beta; NLRP3, NLR family pyrin domain containing 3; p-AMPKα, Phosphorylated AMP-activated protein kinase alpha; PHGG, Partially hydrolyzed GG; SCFA, short-chain fatty acid; TEER, Transepithelial electrical resistance; TJ, Tight junction; TLR, Toll-like receptor; TNF-α, Tumor necrosis factor alpha; Treg, Regulatory T lymphocyte; WAS, Water avoidance stress; YAMC, Young adult mouse colonic cells; ZO, Zonula occludens*.

*In vitro* studies have shown the ability of DF to attenuate epithelial barrier dysfunction caused by bacterial infection (14, 15) (Table 1). Similar *in vitro* studies with SCFAs (Table 2) indicate that main SCFAs, butyrate, propionate, and acetate, modulate contractile activity (40), to maintain the circadian rhythm (47). SCFAs are also able to inhibit cytokine production (16, 49), activate Tregs (45), enhance IEB, by facilitating TJ assembly *via* AMPK activation in Caco-2 cell monolayers and through selective upregulation of claudin (CLDN) 3 and 4, and the activation of Akt/mTOR mediated protein synthesis in IPEC-J2 cells (43), increasing TEER (41–44). Interestingly, the activation of NLR family pyrin domain containing (NLRP) 3 inflammasome induces the secretion of proinflammatory cytokines (46), which is linked to intestinal barrier dysfunction (57). In this regard, a study performed in intestinal epithelium cells IEC-6 showed that propionic acid inhibited NLRP3 inflammasome activation and preserved intestinal barrier function (48).

The role of DF and SCFAs in modulating intestinal barrier function and GI inflammation has been also tested *in vivo* in several preclinical models and in multiple species (Tables 1, 2). In this sense, sodium butyrate has been shown to revert colonic permeability in a rat model of Irritable Bowel Syndrome (IBS) (50). In C57/BL6 mice submitted to chemotherapy-induced mucositis, high fiber diet (pectin-based) decreased the influx of immune cells, improved histopathological parameters and decreased intestinal permeability, compared to those that received the normal diet (58). In obese mice, prebiotics exhibited lower plasma lipopolysaccharide (LPS) and cytokines, and lower intestinal permeability and improved TJ integrity compared to controls (59). Dietary enrichment with psyllium fiber (21), dietary cellulose (20), or lemon peel powder (25) also ameliorated colonic damage and inflammation and decreased TJ protein expression in the dextran sodium sulfate (DSS)-induced colitis model in mice, particularly during the infancy. Sulfated polysachharide not only reduced colonic inflammation, but also but inhibited colon shortening and oedema in mice model (29). In this model, DF also ameliorated intestinal barrier dysfunction and inflammation (51). In specific pathogen-free and germ-free mice given DSS, psyllium, pectin, and cellulose fiber reduced the severity of colitis through microbiota-dependent and microbiota-independent mechanisms, including restoration of intestinal permeability (60). Similar studies in rats have disclosed the ability of high-amylose cornstarch diet to protect against 2,4,6 trinitrobenzene sulfonic acid (TNBS)-induced colonic injury, and improve colonic permeability (17). In the same model, high rich DF containing grape peel powder also reduced colitis and reestablished intestinal barrier function in Wistar rats (24). Moreover, apple-derived pectin has been shown to modulate gut microbiota and CLDN-1 expression in obese rats submitted to high-fat diet, to attenuate metabolic endotoxemia, inflammation, and weight gain (61). In other rat models, the addition of cellulose fiber to elemental diet could ameliorate barrier failure in the ileum compared to total parenteral nutrition (62) and pectin supplementation significantly reversed the methotrexate-induced increase in permeability in the distal small bowel and colon (63).

DF was able to restore colonic barrier integrity in a mice model of chronic kidney disease (22) and GOS administration attenuated intestinal barrier damage and inflammatory responses induced by LPS in the jejunum and ileum of mice (26). In other models, particularly in interleukin (IL)-10 knockout mice with IBD, dietary pectin and cornstarch diets downregulated the inflammatory response in colon, but its relation with the regulation of intestinal permeability was not established (18, 19).

DF, such as Psyllium husk, was able to decrease bowel movements, and improve stool consistency and weight gain in dogs (27). Moreover, Plantago ovata showed effectiveness in increasing body weight and small intestine length as well as in reducing intestinal *E. Coli* in broiler chickens (23). Recent studies have shown that alginate oligosaccharide is able to increase TJ expression, Bacteroidetes, and Firmicutes phylum bacteria and to decrease pro-inflammatory cytokines in weaned pigs (28).

Regarding SCFA, in a rat model of irritable bowel syndrome (IBS) (50), sodium butyrate has been shown to revert colonic permeability. Also in this model, mixed or alone SCFA have been reported improving IBS symptomatology (52). In neonatal IBS-mice model, different concentrations of SCFA were able to reduce the colonic transit alteration in a dose-dependent manner (53).

In humans, conclusions derived from dietary interventions with supplemental fiber have been often inconclusive and weighted down by differences in the design and performance of studies, as highlighted in a recent meta-analysis in IBD population (64). Thus, pectin supplementation (15/day) or daily supplementation (12 g/day) with the DF β-glucan and wheat arabinoxylan did not affect baseline intestinal barrier function in young and elderly healthy individuals (65) or indomethacin-induced intestinal hyperpermeability *in vivo* or gut microbiota composition in elderly, respectively (66). Furthermore, oligofructose-enriched inulin (8 g/day) did not improve intestinal permeability in children with diabetes mellitus (67) as did not either oligofructose (6 g/day) in patients with burn injury (68). Plantago ovata seeds have been shown to maintain remission in UC (30), and FOS increased bifidobacteria and IL-10 in CD patients (31), although it has not been supported by further research (32). However, CD patients were shown to achieve an improvement in their quality of life and GI function after wheat bran intake (33). In other study, healthy male volunteers who ingested inulin for 8 weeks (69), had significantly lower lactulose/mannitol (L/M) ratio and serum zonulin and higher levels of mucosal GLP-2. However, it is important to note that the methods used were suboptimal. On the contrary, a non-digestible polysaccharide-enriched diet reduced colonic hyperpermeability induced by mast cell (MC) activation, as determined in Ussing chambers, in elderly suffering constipation or diarrhea and elevated baseline colonic permeability (35). Similarly, in patients with non-alcoholic fatty liver disease, 6-months of fiber intervention demonstrated a reduction in zonulin levels, a purported serum biomarker of permeability (36), and GOS supplementation reduced aspirin-enhanced colonic permeability in obese patients independently of its prebiotic effect (70). The combination of green banana and pectin showed good antidiarrheal properties in children with persistent diarrhea, activity that was linked to the reduction in small intestinal permeability (71).

SCFAs are also able to modulate intestinal permeability in humans. Indeed, decrease in gut-derived plasma SCFAs correlated with increased colonic permeability in shift workers (72) and organoid studies based on human colonic mucosal biopsies showed that fermentation of 2′ Ofucosyllactose which led to an increase of Bifidobacteria and an increase of SCFAs, in particular butyrate, resulted in CLDN-5 significant upregulation (73).

A diet low in fermentable oligosaccharides, disaccharides, monosaccharides, and polyols (FODMAP) is commonly used in the management of patients with IBS and overall, 52–86% of patients report significant improvement of their symptoms (34, 37, 74). Moreover, this diet was more effective than others (traditional dietary advice, modified National Institute for Health and Care Excellence guidelines, gluten-free diet and Mediterranean diet and a sham diet) and also non-dietary interventions (gut directed hypnotherapy or yoga) (75). Interestingly, this diet improved intestinal permeability in patients with diarrhea-predominant IBS in relation with increased circulating vitamin (vit) D levels (76). However, to date, there is no further demonstration of how low-FODMAP diet may interfere with intestinal permeability.

Recently, a combination of herbs and nutrients including curcumin, aloe vera, slippery elm, guar gum, pectin, peppermint oil, and glutamine (Gln) significantly improved the frequency and severity of upper and lower GI symptoms by 60–80% in a small sample sized study. This improvement was accompanied by reduction of intestinal permeability, as measured by lactulose-mannitol ratios, and by beneficial changes in microbiota composition (77).

Other dietary factors such as the non-sugar prebiotics soy protein hydrolysates have shown promising effects to strengthen the epithelial barrier in response to several barrier disruptors (78).

#### Vitamins

Vit A, and vit D are micronutrients involved in the regulation of TJ molecule expression in the intestinal barrier (79) and mucosal immune system, shaping the microbial populations in the gut (80, 81). Both epithelial and immune cells in the GI tract, but not the microbiota, express receptors for vit A (retinoic acid receptor) and vit D (vit D receptor, VDR) (4). VDR protects against mucosal inflammation in experimental colitis and contributes to systemic bile acid homeostasis by regulating expression of fibroblast growth factor (82). Retinoic acid receptor enhances Zonula Occludens (ZO)-2 expression by regulating Toll-Like Receptor (TLR)-4 to improve IEB function in Caco-2 cells, as well as in rat and mouse models, but not in humans (83).

The presence of vit D increased TEER and preserved the structural integrity of the TJ in Caco-2 cells treated with DSS (84). In a model of intestinal barrier permeability using IPEC-J2 cells, vit A reverted LPS-induced barrier dysfunction through the enhancement of TEER and TJ protein expression (85). In Caco-2 cells treated with LPS, emulating the barrier damage of necrotizing enterocolitis (NEC), the presence of 1,25-Dihydroxyvitamin D3 -active form of vit D- restored the expression and localization of TJ proteins and reverted LPS-induced down-regulated VDR expression (86). Likely, intestinal damage caused by LPS in IEC-18 line cells and organoids was improved after vit D treatment, restoring permeability and TJ (87). Similar findings have been reported in a model of alcoholic liver disease in Caco-2 challenged with ethanol (88). Moreover, vit D deficiency may compromise mucosal barrier integrity, raising susceptibility to develop IBD, as also shown in Caco-2 cells (89).

In specific-pathogen-free rats, the deficiency of vit A aggravates the severity of diarrhea and intestinal mucosal damage. On the contrary, during the clinical course of diarrhea, supplementation with vit A relieves diarrhea and improves intestinal damage, increasing the expression of TJ proteins (90). When intestinal epithelial cells from VDR-deficient mice are complemented with a human VDR-encoding trans-gene, the integrity of the mucosal barrier prevents the hyperinflammatory response that is otherwise seen in the lamina propria immune cells of VDR-deficient mice (91).

In humans, several reports indicate insufficient levels of vit D in many inflammatory conditions, including IBD (92, 93) and IBS (94), with more than 50% of patients affected by hypovitaminosis (95, 96). Vit D deficit has been also related with clinical symptoms and quality of life, but the correlation between the intestinal expression of VDR and CLDN2 remains controversial (97–100). Patients with celiac disease (CD) in remission received a supplementation of vit D -or placebo- during 3 months. The supplemented patients showed higher plasma levels of vit D, improved the results of quality-of-life and kept intestinal permeability as it was at baseline, whereas permeability increased in the placebo group (101). As previously mentioned, a recent study assessed the relation between vit D levels, intestinal permeability, and a 12-week intervention with low-FODMAP diet in IBS with diarrhea (IBS-D). Those patients with low vit D levels before the intervention, reduced small bowel permeability, increased vit D levels and also improved clinical symptoms (76).

#### Amino Acids

The pore pathway regulates, through IL-13-mediated expression of CLDN-2, the selective paracellular transport of smalI-sized (5–10 Å) ions (K+, Na+) and molecules (water) (102, 103). *In vitro* studies in Caco-2 cells with deprived from Gln or after Gln synthetase inhibition, reported reduced TEER, increased permeability and lower TJ protein expression (104–106) which could be reestablished after Gln addition. Gln, and to a lesser extent arginine (Arg), also prevented methotrexate-induced barrier disruption in Caco-2 cells (107). Gln improved intestinal barrier function in a rat model of biliary obstruction (108), and Gln and Arg prevented the mucosal injury in a model of ischemia-reperfusion in rats (109, 110). Gln regulated TJ integrity and distribution through calcium/calmodulin-dependent kinase 2 (CaMKK2)-AMP-activated protein kinase signaling in porcine epithelial cells (111). Recently, Gln alleviated IL-13-induced barrier dysfunction by increasing CLDN-1 expression, *via* disruption of the phosphatidylinositol-3-kinase/Akt signaling pathway (112).

In humans, microRNA (miR)-29 has been shown to regulate Gln synthetase, CLDN-1 expression, nuclear factor kappa-light-chain enhancer of activated B cells (NF-κB) and ultimately tumor necrosis factor (TNF-α), to regulate the leak paracellular pathway in a series of elegant experiments performed in colonic tissues of IBS-D patients (113). Moreover, in a subsequent randomized, placebo-controlled trial, the same authors showed that supplemental Gln (10 g/day) improved intestinal permeability and major symptoms in post-infectious IBS-D patients (114). In addition, although disputed, enteral Gln supplementation has been shown to improve intestinal permeability in severely thermally injured patients (115). A small randomized trial also showed that Gln and whey protein improved small intestinal permeability and morphology in patients with Crohn's disease (116).

Arg is a semi-essential amino acid that can be metabolized by host arginases and nitric oxide synthases or be consumed by gut bacteria (4). There are few studies related to Arg and protection of the integrity of the epithelial barrier. In heat-stress conditions, pre-treatment with L-Arg partly reverted the decrease on TEER and increased paracellular permeability (117). In a model of hypoxia in jejunal IPEC-J2 cells, Arg prevented the reduction of TEER and increased inulin paracellular permeability (118). A great compilation of the last 30 years of clinical trials performed with Gln and Arg is also available (119), highlighting a reduction of the infection rate and mortality by Gln and a decrease of complications by Arg in surgical patients. Nevertheless, some of these trials have methodologic flaws and many do not evaluate intestinal permeability. Hence, further and well-designed trials are needed for justifying the use of these amino acids.

Tryptophan is an essential amino acid also studied by its potential link between imbalanced gut microbiota, impairment of intestinal immunity and disease development. Recent evidence underlines that the enzyme indoleamine 2,3-dioxygenase 1 expressed by the host is relevant to generate indole metabolites (120), which are involved in the re-establishment of IEB integrity in the context of intestinal inflammatory diseases and metabolic syndrome.

A recent review on amino acid supplementation in weaned piglets, disclosed that several of them (Arg, Gln, tryptophan, sulfur-containing amino acids, and branched-chain amino acids) may have a role in the maintenance and improvement of intestinal morphology and function, increasing proliferation of epithelial cells and preserving intestinal mucosal integrity (121). In mice, radiation-induced intestinal barrier disruption was ameliorated by an amino acid-based oral rehydration solution, enhancing TJ protein expression and improving paracellular permeability (122).

#### Minerals

Zinc (Zn) is an essential trace element [10% of the human genome encodes Zn-binding proteins (123) that plays an important role in diarrheal diseases and GI infections and it is closely linked to mucosal integrity and IEB (124). Zn deficiency leads to reduced expression of occludin (OCLN) and ZO-1 proteins in Caco-2 cells (125). Depletion of Zn induced OCLN-3 proteolysis and decreased CLDN-3 transcription (126) while Zn supplementation increased TEER and ZO-1 expression and decreased CLDN-2 and CLDN-7 expression (127, 128), facilitating OCLN and ZO-1 expression in Caco2 and HT29 cells (90). In mice with bacterial infections, Zn supplementation enhanced protection against toxin-induced intestinal dysfunction and leakage (129). The ZRT/IRT-like protein 14, Zn transporter is expressed on plasma membranes and mediates Zn influx into the cytosol. Mice lacking ZRT/IRT-like protein 14 display increased intestinal permeability associated with altered expression of CLDN-1 and CLDN-2 (130). Other studies have shown that Cu and Zn supplementation improved intestinal integrity during the *Eimeria* spp. Infection in broilers (131). Selenium has also been proposed as a good candidate to prevent changes in intestinal permeability and mitochondrial damage in several species (132, 133). In humans, Zn supplementation is effective in the prevention of diarrhea (134), and has been recommended by The World Health Organization for the treatment of diarrhea (135). Zn also has a beneficial effect on infectious diseases like shigellosis improving IEB, nitrogen absorption, and symptoms (135–137). Finally, zinc carnosine, a health food supplement, stabilizes small bowel integrity and stimulates gut repair processes after indomethacin treatment, as shown in a placebo-controlled trial (138).

### Microbiota-Based Factors

Microbiota exerts many crucial functions (thoroughly reviewed in other papers in this monography) including IEB maintenance (139, 140).

#### Antibiotics

Antibiotics are recommended to treat bacterial infections. Independently of the origin of the infection, antibiotic administration has adverse effects on the gut indigenous microbial community leading to mid to long term dysbiosis (141) and mycobyosis (142), with some compositional effects lasting for 6 months (143), to ease colonization by pathogens such as *Salmonella* or *Cl. Difficile*, and to increase antibiotic resistance (144, 145). In addition, many antibiotics induce changes in intestinal permeability that may be linked to alterations in α-diversity and relative abundance of specific bacteria within the gut microbiota as shown in rats (146). Moreover, changes in intestinal permeability are accompanied by reduction of SCFAs, and increased activity of NLRP3 inflammasome and autophagy (147). Therefore, the use of some antibiotics in disorders associated with barrier dysfunction may lead to additional complications, though these findings should be translated to the clinic. In addition, bioavailability of antibiotics seems to depend also on the composition of microbiota and on intestinal permeability as well (148).

However, some antibiotics may have a better profile for the microbiota. Rifaximin is a poorly-absorbed broad spectrum oral antibiotic prescribed for GI disorders such as IBS, IBD, small intestine bacterial overgrowth, traveler's diarrhea or diverticular disease (DD) (149, 150). Rifaximin seems to exert eubiotic effects on the microbiota, increasing *Bifidobacterium, Faecalibacterium prausnitzii*, and *Lactobacillus* abundance, with no major change in the overall gut microbiota composition, what may represent a therapeutic advantage in specific clinical settings (150, 151). In addition, Xu et al. (152), showed how oral rifaximin prevented mucosal inflammation, impairment to intestinal barrier function, and visceral hyperalgesia by altering the composition of bacterial communities in the ileum while other antibiotics were not as effective.

#### Probiotics

Probiotics are live microorganisms which, when consumed in adequate amounts, confer a health benefit on the host (153). This benefit relates in part to the ability of probiotics to modulate the IEB. A large amount of evidence has accumulated to support the efficacy of probiotics to enhance IEB tightness and integrity, and to modulate intestinal inflammation (154). We will only review here the most representative evidence. For instance, *Bifidobacterium* was able to adhere to mucus, to inhibit and displace the adhesion of pathogenic bacteria (155) and to increase TJ integrity, protecting them from *Escherichia coli* O157:H7 (156). Incubation of T84 cell monolayers with multispecies probiotic completely prevented LPS-induced increase in paracellular permeability in a dose dependent manner. This multispecies probiotic also prevented the epithelial disruption induced either by intracolonic infusion of fecal supernatant from IBS patients or by water avoidance stress (WAS) in C57/Bl6 mice. In addition, these formula increased the expression of OCLN and decreased TNF-α secretion in response to LPS (157). Similarly, *Lactobacillus rhamnosus* CNCM I-3690 prevented changes in intestinal permeability in Caco-2 cells stimulated with TNF-α and in a mouse model of increased colonic permeability, to a similar degree that *Faecalibacterium prauznitzii* A2-165 in the last (158). In a post-infectious IBS mouse model, probiotic treatment promoted the expression of major TJ proteins CLDN-1 and OCLN in the mouse ileon (159). Similarly, *Lactobacillus rhamnosus* GG improves intestinal barrier function in the immature murine gut through the induction of CLDN 3 expression (160). In obese and type2 DM mice, *Akkermansia. muciniphila* treatment increased the intestinal levels of endocannabinoids that control inflammation and gut barrier (161).

Probiotics can also prevent intestinal barrier damage in IBD conditions. Both LGG and a probiotic formulation containing *Lactobacillus acidophilus, Bifidobacterium lactis, Lactobacillus plantarum*, and *Bifidobacterium breve* reduced the disruption of barrier function in DSS-induced colitis in mice (162, 163). In a similar way, the administration of a probiotic mixture prevented not only the decrease in TJ proteins expression, but also the increase of epithelial apoptotic ratio induced by acute colitis (164). Oral *Bifidobacterium infantis* conditioned medium administration reduced colonic permeability in IL-10-deficient mice in part through enhanced protein expression of CLDN-4, ZO-1, and OCLN, and decreased expression of CLDN-2 (165). Similarly, *Escherichia coli Nissle* 1917 has been shown to inhibit leaky gut by enhancing mucosal integrity through up-regulation of ZO-1 expression in murine DSS colitis (166). *Lactobacillus rhamnosus* MTCC-5897 administration before DSS-colitis induction improved intestinal barrier integrity involving transcriptional modulations of TJ genes (ZO-1, OCLN, CLDN-1) (167).

Stress clearly affects intestinal barrier function and probiotics have been shown to prevent some of changes. *Weissella paramesenteroides* WpK4 ingestion reduced intestinal permeability and reduced anxiety-like and depressive-like behaviors in stressed mice submitted to DSS (168). *Lactobacillus farciminis* prevented stress-induced gut hyperpermeability and mucus alterations in different animal models (169, 170). In rats subjected to partial restraint stress fermented milk containing *Bifidobacterium lactis* CNCM I-2494 prevented stress-induced increase in intestinal permeability and restored OCLN and JAM-A expressions to control levels (171). More recently, Wang et al. have shown that *Lactobacillus casei* Zhang significantly increased jejunum villus height, villus height-crypt depth ratio, muscle thickness, and expression of proliferating cell nuclear antigen and TJ proteins ZO-1 and OCLN in early-weaned piglets, and prevented *E. coli* K88-induced jejunum damage (172). Similarly, *Lactobacillus fermentum* CECT 5716 prevented maternal separation and WAS-induced intestinal barrier dysfunction in newborn rats, reducing small intestine permeability and increasing ZO-1 expression (173).

In humans, *Lactobacillus plantarum* WCFS1 administration into the duodenum was associated with an increase in of ZO-1 and OCLN in healthy subjects (174). In contrast, *Lactobacillus* GG significantly reduced the alteration of gastric (but not intestinal permeability induced by indomethacin administration in healthy subjects suggesting that probiotics are useful to enhance barrier function possibly in a location-specific manner (175).

*Bifidobacterium lactis* CNCM I-3446 induced a significant decrease of intestinal permeability in infants with NEC (176). In IBD patients, particularly in those with severe pouchitis, administration of a probiotic combination effectively prevented flare-ups (177), combination that has been shown to promote recovery from IFN-γ-induced intestinal barrier dysfunction (178).

Recently, beneficial effects of probiotics have also been shown to occur through the release of extracellular vesicles (EV). EV contain a vast number of active compounds and bacterial mediators that play a key role in bacteria-host interactions, but also between probiotics and other bacteria. *In vitro*, pretreatment with *Akkermansia muciniphila*-derived EV decreased IL-6 production from colon epithelial cells stimulated by pathogenic *Escherichia coli* EV (179). *Akkermansia muciniphila*-derived EV (179) also protected from DSS-induced colitis in C57BL/6 mice. Moreover, in high-fat diet-induced diabetic mice, *Akkermansia muciniphila*-derived EV administration enhanced tight junction function, reduced body weight gain and improve glucose tolerance in association with an increase in the expression of occludin, zonula occludens, and claudin-5 (180). In fact, in the same study, more *Akkermansia muciniphila*-derived EV were found in the fecal samples of healthy controls when compared with type 2 diabetes patients and *Akkermansia muciniphila*-derived EV treatment improved intestinal permeability in LPS-treated Caco-2 cells, by increasing the expression of occludin.

Vesicles secreted by *Bacteroides fragilis* have been shown to contain capsular polysaccharide, which induces immunomodulatory effects on dendritic cells and prevents experimental colitis through TLR2-signaling pathways (181). In Caco2 cells, *B. fragilis* EV significantly decreased TLR2 and slightly increased TLR4 mRNA levels along with an increase in anti-inflammatory cytokines and the inhibition of interferon (IFN)-γ (182). Stimulation of bone marrow derived dendritic cells (BMDCs) with EV originated from another Gram-negative probiotic, *Bacteroides vulgatus mpk*, contributes to immune response silencing through induction of a tolerant BMDCs phenotype (183).

*Escherichia coli* Nissle 1917 (EcN) has also been shown to restore barrier function in experimental models of increased gut barrier permeability. Prophylactic administration of EcN resulted in reduced inflammation, and preservation of intestinal permeability in a DSS murine model of colitis (184). EcN treatment significantly upregulated the colonic expression of the tight junction proteins ZO-1 and occludin, preserving the mucus- layer and restoring intestinal permeability. Oral administration of purified EcN EV before DSS intake, significantly reduced clinical symptoms and histological scores in a DSS-induced colitis mouse model (185). Similarly, in colonic cell lines, EcN EV promoted upregulation of ZO-1 and claudin-14, and induced IL-22 expression reinforcing the intestinal barrier (186).

However, few studies have specifically addressed intestinal permeability *in vivo* in humans. One study assessed the efficacy of a probiotic mix in intestinal permeability, immune function and in the prevention of multiple organ dysfunction syndrome in critically ill patients. They found that patients responded with a significantly larger increase in systemic IgA and IgG concentrations and in most of them, intestinal permeability decreased, compared to placebo or sonicates (187). Mujagic et al. evaluated the effects of *Lactobacillus plantarum* on small intestinal barrier function through the lactulose-rhamnose ratio after intake of indomethacin, but there was no significant effect. However, in small intestinal biopsies, *L. plantarum* TIFN101 modulated gene transcription pathways related to cell-cell adhesion with high turnover of genes involved in tight- and adhesion junction protein synthesis and degradation (188). A recent meta-analysis, evaluated the effect of probiotics/synbiotics on serum levels of zonulin, as a measure of intestinal permeability, showing favorable effects although results should be interpreted with caution due to high heterogeneity (189). Another recent meta-analysis highlights also the potential beneficial role of probiotics in GI mucositis and the reduction of intestinal permeability and maintenance of the mucus layer (190).

Taken together, all these data suggest that probiotics enhance intestinal barrier tightness and integrity by several mechanisms, and that mucosal restoration positively impacts the clinical course of disease. However, specific studies measuring intestinal permeability through a validated method are needed to achieve more robust conclusions.

### Bioactive Pharmaceutical Molecules and Signaling Peptide-Based Therapeutic Strategies

#### Glucagon-Like Peptide 2

**Figure 3:**
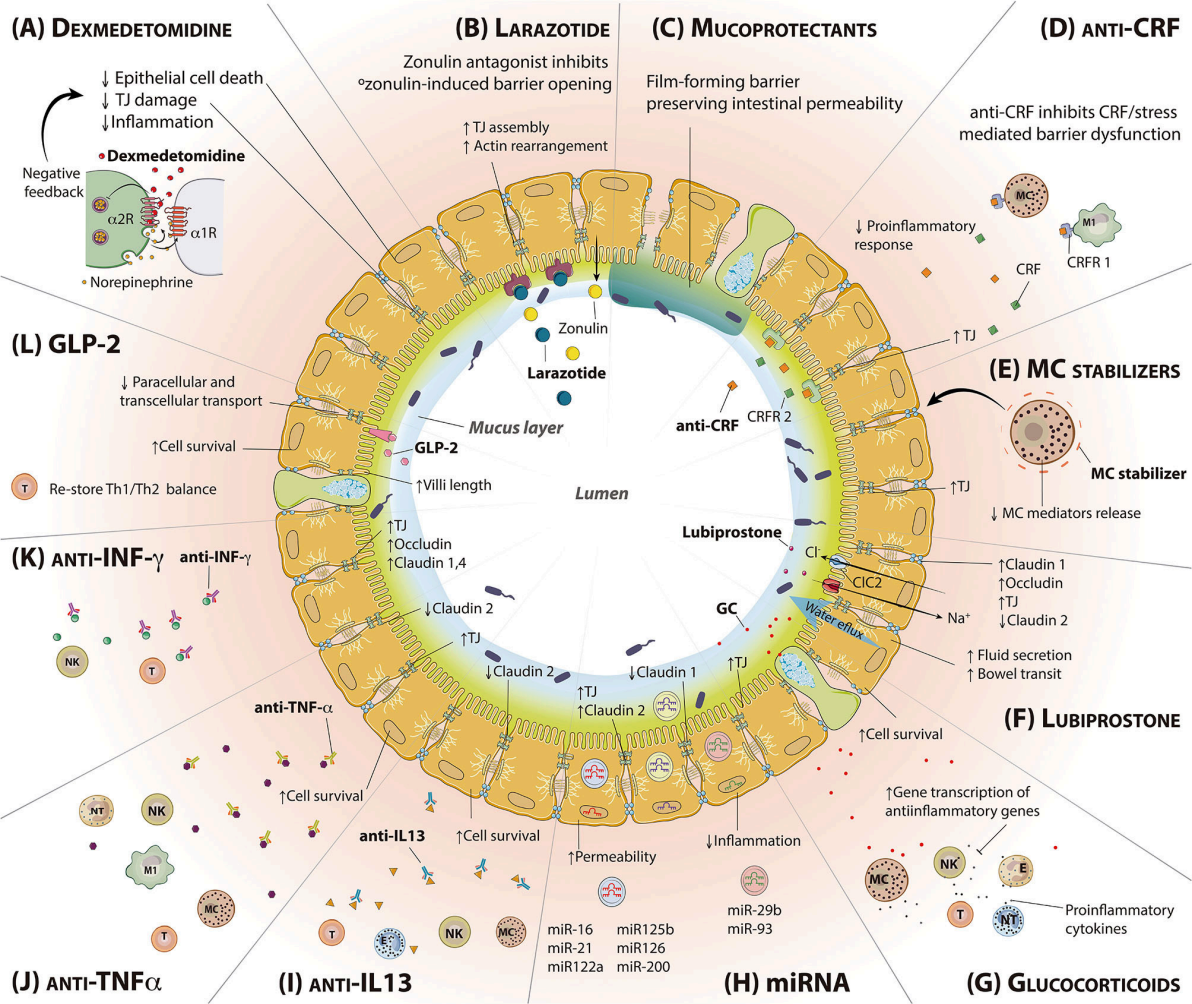
Molecules intended to regulate intestinal permeability and/or mucosal inflammation. Representation of a transversal section of the small intestine, including epithelial and goblet cells. The mucus layer and the lumen are found inside; the *lamina propria* is outside. **(A)** Dexmedetomidine reduces epithelial cell death, TJ damage and inflammation. **(B)** Larazotide is a zonulin antagonist, able to bind zonulin receptor and block its toxic effects. **(C)** Mucoprotectants cover the epithelial cells surface forming a film barrier that helps to preserve intestinal permeability. **(D)** CRF antagonist binds to CRF receptors blocking the binding of stress-released CRF and decreasing subsequent proinflammatory responses. **(E)** MC stabilizers are responsible for MC cell membrane stabilization inhibiting degranulation. **(F)** Lubiprostone increases water flux to the intestinal lumen and bowel transit, enhancing the expression of TJ proteins. **(G)** Glucocorticoids inhibit the activation of the immune system via transcription of anti-inflammatory genes. **(H)** miRNA exert different roles within the epithelial barrier regulation, being able to enhance the intestinal permeability or reducing inflammation **(I)** Anti-IL-13 treatment inhibits IL-13 effects in the IEB, increasing cell survival and decreasing the expression of the pore-forming TJ claudin 2. **(J)** Anti-TNF-α increases cell survival and TJ expression while decreases claudin 2 expression through TNF-α antagonism. **(K)** Anti-INF-γ treatment inhibits INF-γ effects in the IEB, increasing the expression of TJs. **(L)** GLP-2 binds to GLP-2R, predominantly expressed in the intestinal tract, resulting in an increase of cell survival and villi length, restoring Th1/Th2 balance. CRF, Corticotropin-releasing factor; CRFR, Corticotropin releasing factor receptor; GC, Glucocorticoids; GLP-2, Glucagon-like peptide 2; GLP-2R, Glucagon-like peptide 2 receptor; IEB, Intestinal epithelial barrier; IL-13, Interleukin 13; INF-y, Interferon gamma; LBP, Lubiprostone; MC, Mast cell; TJ, Tight junction; TNF-α, Tumor necrosis factor alpha.

Glucagon-like peptide 2 (GLP-2) (Figure 3) is an intestinal peptide derived from proglucagon that exerts its function through the GLP-2 receptor (GLP-2R), expressed predominantly in the intestinal tract (191, 192). Endogenous GLP-2 promotes intestinal growth after a fasting period or in response to enteritis (193). Exogenous GLP-2 is exerts profound effects expanding the crypt-villus epithelium through enhanced proliferation and survival, to increase nutrient digestion, absorption, and blood flow (192, 194, 195).

GLP-2 also improves intestinal barrier function in both health conditions and disease models (59, 196, 197), reducing paracellular transport of ions and small molecules, and dramatically inhibiting endocytic macromolecules uptake in mice (197). GLP-2 chronic administration enhances gut barrier function and decreases epithelial barrier permeability. In fact, GLP2 in mice model decreases the transcellular passage of ions, 51Cr-EDTA and fluorescein-isothiocyanate as well as the endocytosis of horseradish peroxidase (HRP), a marker of transcellular permeability (198–200). Following studies have demonstrated that this ability is GLP-2R-dependent, in association with increased TJ expression, most notably CLDN-3 and−7 (196, 199). In addition, rats receiving subcutaneous exogenous GLP-2 exhibited less intestinal structural damage, longer intestinal villi, and increased immunoglobulin (Ig)A expression, in a model of obstructive jaundice (201).

It has been widely reported the GLP-2 effect on increasing microvillus length, however, how this is achieved is poorly understood. Recently, Markovic et al. (202) demonstrated that the increase in microvillus length with GLP-2 treatment requires the intestinal epithelial insulin-like growth factor-1 receptor (IE-IGF-1R) in mice. Villin, an actin-binding protein, is regulated by the GLP-2-IE-IGF-1R pathway. Villin has a well-established role in epithelial wound repair, with both insulin growth factor 1 and villin levels decreased in Crohn's disease (203, 204). These findings suggest a new mechanism by which GLP-2 may attenuate Crohn's disease and/or other inflammatory pathologies. These results are consistent with previous research which has already shown the effects of GLP-2 through the IE-IGF-1R modulating intestinal TJ proteins (199). In this regard, a study in pediatric patients with acute ileal CD showed that these patients have decreased post-prandial GLP-2 release, and increased intestinal permeability. Healing of CD was associated with the normalization of post-prandial GLP-2 release and intestinal permeability (205). More recently, an abnormal post-prandial glucagon-like peptide 2 release has also been described in adult patients with Crohn's disease (206).

Finally, GLP-2 analog teduglutide has been successfully introduced in clinical practice as a new treatment for parenteral nutrition-dependent short bowel syndrome (207). It can effectively increase the residual intestinal absorption capacity through the induction of intestinal mucosa hypertrophy and hyperplasia, the increase of intestinal perfusion and the reduction of intestinal motility and gastric acid secretion, achieving a reduction of parenteral nutrition (208, 209). Several cases of active Crohn's disease and short bowel syndrome successfully treated with teduglutide have been recently described (210–212). Yet, there are no systematic data about off-label teduglutide therapy in severely active CD since its fluctuating inflammatory activity can be considered at greater risk (213).

All these findings support GLP-2 treatment as a possible effective therapy for enhancing, maintaining, or recovering normal barrier function in intestinal disorders. However, to date, human studies evaluating the effect of GLP-2 on intestinal permeability do not exist. Nevertheless, teduglutide must be used with caution and discontinued in case of intestinal neoplasia because of its effect on intestinal epithelial proliferation.

#### Corticotropin-Releasing Factor

Corticotropin-releasing factor (CRF) is a signaling peptide (Figure 3), secreted both in the central nervous system and in the periphery, including the GI tract, which stimulates the secretion of adrenocorticotropic hormone from the pituitary gland in response to stress. CRF and related molecules such as urocortins 1, 2, and 3 have been extensively involved in the regulation of stress-mediated motor, sensory and permeability changes in the GI tract, in animal models and humans, *acting via* the G-Protein coupled CRF receptors (CRF-R) 1 and CRF-R2 (214–217).

Many authors have described the effects of stress on gut permeability. Studies in rats and pigs have shown that CRF-induced changes over the barrier function were equivalent to those triggered by stress. Among other alterations, CRF induces mucus layer thickening, enhanced conductance and transepithelial and paracellular macromolecular flux, TJ reorganization and activation of the immune system in the small intestine and colon (218–224). Consistent evidence indicates that many of these mechanisms are predominantly driven by the activation and degranulation of MCs (221, 223–225), although recent studies lay stress on eosinophils as potential contributors to the stress-mediated gut dysfunction, specifically in IBS-D patients (226). Opposite effects of CRF-R1 and CRF-R2 are observed on stress-mediated intestinal mucosal barrier function in pigs, with CRF-R2 preventing permeability changes and CRF-R1 enhancing them (227).

Interestingly, chronic stress has shown how barrier impairment could be persistent if the stressor is repeated (222, 228–230). Vicario et al. reported increased gut epithelial permeability, hyperactivation of the hypothalamic-pituitary-adrenal axis and reversible inflammation in rats submitted to a repeated stress or CRF, developing visceral hypersensitivity afterwards (230). Similarly, CRF and sauvagine, a stress-like peptide, enhanced intestinal ion, and macromolecular flux, which could be inhibited by astressin, a potent non-specific CRF inhibitor, and doxantrazole, a MC stabilizer. The alterations of intestinal permeability evoked by various stressors or CRF are inhibited by peptide CRF receptor antagonists and selective CRF-R1 antagonists (219, 221–224, 226, 228–232) [for further review, see Taché et al. (217)]. Moreover, Nozu et al. have recently reported in a rat IBS model that imipramine dose-dependently inhibited visceral hypersensitivity, colonic hyperpermeability, and other GI effects of CRF or repeated stress through α2-adrenoceptors, dopamine and opioid receptors (233).

In healthy humans and IBS patients, functional studies also demonstrated that peripheral CRF largely reproduces the increased colonic motility, intestinal permeability, MC activation and visceral hypersensitivity observed in animals (224, 234). Changes in intestinal and colonic permeability were mediated by MC activation and reversed by disodium cromoglycate, another MC stabilizer (235). Nonetheless, despite raising high expectation early on, and several clinical assays performed with several CRF antagonists (236–240), unfortunately, to date, this has not been translated in clinical practice for the management *of stress-induced IBS*.

#### Humanized Antibodies Against Tumor Necrosis Factor-α

Tumor necrosis factor-α (TNF-α) (Figure 3) and myosin-light chain kinase (MLCK) are the main regulators of the leak paracellular pathway (102). TNF-α is a multifunctional pro-inflammatory cytokine that has wide effects on cells and structures related to the intestinal barrier function. One of the barrier-deteriorating effects likely comes from TNF-α's ability to induce apoptosis (241). However, TNF-α was found to deteriorate paracellular integrity even in the presence of an apoptosis blocker, suggesting additional mechanisms involved. Indeed, *in vitro* studies found that TNF-α modulates TJ structure by breaking strands of ZO-1 and thus modifying the structure of the epithelial barrier (242). TNF-α stimulation could also increase permeability by inducing the expression of the pore-forming TJ protein CLDN-2 (243). There is also evidence for TNF-α increasing paracellular permeability by activating long MLCK transcription, expression, enzymatic activity, and recruitment to the actomyosin ring (244–247). MLCK activation triggers perijunctional actomyosin ring contraction that leads to molecular reorganization of TJ structure, including OCLN endocytosis.

The use of monoclonal antibodies against TNF-α has shown mixed results, some proven successful in inducing remission in cases of IBD (248), but also at the cost of adverse events and high number of non-responders over time (249, 250). Few but promising evidence suggest targeting TNF-α can improve specific parts of the intestinal barrier function and endoscopic signs on the mucosal tissue, collectively termed mucosal healing (251).

Adalimumab is a monoclonal antibody against TNF-α shown to improve both IEB and clinical features in IBD patients (252–256). Human colonic cell culture showed that simultaneous use of both TNF-α and interferon (IFN-γ) disrupted the epithelial barrier, leading to a significant drop in TEER (257), appearance of irregularities in the TJ structures, disruption of OCLN and increase phosphorylation of MLC. All of these effects were reversed upon administration of Adalimumab. When subjecting a 3D Caco-2 cell model to plasma from patients with active Crohn's disease, paracellular permeability increased *via* breakdown of ZO-1 and OCLN (258).

Infliximab, a chimeric monoclonal antibody against both membrane bound and soluble TNF-α, has shown successful results in both patients with Crohn's disease and UC (256, 259–261). Crohn's disease patients display an increased baseline permeability compared to healthy controls, that was normalized after 7-days course of infliximab, for a final 10-fold decrease of the lactulose/mannitol ratio. The effect of Infliximab on barrier function was also investigated by mounting non-inflamed colonic biopsies from Crohn's disease patients in Ussing chambers and in Caco-2 cells. The results showed a significant decrease in paracellular permeability and normalization of transmucosal permeability to near control levels for adherent invasive *Escherichia coli* (262). Efficacy of infliximab has not been well-studied for other conditions characterized by intestinal barrier dysfunction. However, anti-TNF-α therapy is a common rescue medication for diarrheal conditions refractory to steroid therapy, including immune-related diarrhea after immune checkpoint inhibitor therapy (263) or microscopic colitis (264). A case-study of a refractory CD patient showed improvement in symptoms and intestinal histology after Infliximab treatment, suggesting a possible effect on the barrier function (265).

Inhibition of MLCK expression or enzymatic activity results in systemic toxicity making these molecules unsuitable as therapeutic targets for barrier control. However, recently a new molecule, termed divertin prevents MLCK1 recruitment to the acto-myosin ring without inhibiting enzymatic function. In this way, divertin restores TNF-induced barrier dysfunction and prevents disease progression in experimental chronic IBD (266).

Collectively, these results indicate that mucosal healing and clinical remission in IBD patients may be strongly related to the immunomodulatory effects from blocking TNF-α, with improvements to the intestinal barrier function occurring as a secondary effects that synergistically improve the outcome.

#### Interferon-Gamma (IFN-γ)

Interferon type II (IFN-γ) is widely known as a pro-inflammatory cytokine with potent effects on intestinal barrier function (267) (Figure 3). Studies performed *in vitro* have found IFN-γ to influence paracellular permeability by affecting structural properties of the epithelial barrier. It has been shown in colonic T84 cell lines that IFN-γ can internalize the TJ proteins OCLN, CLDN-1, CLDN-4 and junctional adhesion molecule A, thereby decreasing TEER and increasing the passage of paracellular markers (268, 269). The internalization process was found to involve cytoskeletal contraction in a MLCK-independent manner (269), which separates it from the mechanisms of TNF-α through more direct effects on barrier integrity, even though the end result is similar. Modern *in vitro* techniques using intestinal organoids (3D cell culture models) have further verified the ability of IFN-γ to disrupt the epithelial barrier function through TJ protein degradation and delocalization (270). A number of studies show a synergistic deleterious effect on intestinal barrier function from the combination of IFN-γ and TNF-α (267, 271). One of the mechanisms behind this synergistic effect could come from IFN-γ's ability to increase the expression of TNF receptor-2, as shown by restoration of barrier function when blocking TNF receptor-2 but not TNF receptor-1 (272). Viceversa, TNF-α has also been demonstrated to increase the IFN- receptor expression *in vitro* (16426148). IFN-γ is seen increased in many intestinal conditions that also are characterized by gut barrier dysfunction, such as IBD and IBS (273, 274). Despite several attempts to create antibodies for IFN-γ or its receptors (275), clinical applicability is difficult due to its ubiquity in cells and organs and its pleiotropic effects. Anyhow, a monoclonal antibody against IFN-γ, AMG 811, is under development (276).

#### Humanized Antibodies Against Interleukin-13

IL-13 is a cytokine extensively involved in inflammatory reactions and mainly produced by T helper-2 cells, MCs, eosinophils, and natural killer cells (277) (Figure 3). The effect of IL-13 on barrier function has not been widely studied but *in vitro* experiments using colonic epithelial cell lines have shown upregulation of the pore-forming TJ protein CLDN-2 together with an increase in paracellular permeability (278, 279). Activation of MLCK can lead to an increased production of mucosal IL-13 together with an upregulation of CLDN-2 in mice (279). Further on, IL-13 also shares with TNF-α the ability of inducing epithelial apoptosis and this effect can be enhanced by the stimulation of both cytokines simultaneously, hinting at a possible synergistic effect (280).

An increased expression of IL-13 has been found in the lamina propria mononuclear cells from Crohn's disease and ulcerative colitis (UC) patients (281). In the same study, stimulation with IL-13 displayed a decreased TEER in a cell culture model of HT-29/B6 cells and by an increase in the pore-forming TJ CLDN-2, while levels of both ZO-1 and OCLN were unaffected. In addition, they also found significantly increased permeability of the sugar probes lactulose and mannitol, and higher rate of apoptosis *in vitro* (281). Although information on mucosal IL-13 levels in patients with IBS is scarce, there are results showing serum levels of IL-13 being significantly increased in UC patients with IBS-like symptoms (282). However, the role of IL-13 in the pathophysiology of inflammatory intestinal disorders is controversial as later clinical studies with monoclonal antibodies against IL-13 (tralokinumab, anrukinzumab) in UC fail to report convincing results (277, 283). These studies did not investigate any direct parameters of intestinal barrier function such as permeability or TJ gene/protein expression, thus it's possible that the anti-IL-13 agents could have had affected such parameters, mimicking the *in vitro* studies, but to an ineffective degree. The anti-IL-13 agent lebrikizumab seems to have positive effects on patients with atopic dermatitis, a chronic inflammatory skin condition characterized by skin-barrier defects. Even though the mechanisms behind improvement could speculatively be linked to restoration of skin barrier function, to our knowledge, no studies have yet elucidated such mechanisms (284, 285). It is likely the anti-inflammatory effects from inhibiting IL-13 indirectly also could help to maintain barrier integrity to some extent. However, these results do not seem to suggest targeting only IL-13 is an effective option in treating conditions of intestinal barrier dysfunction. Since multiple cytokines can have deleterious effects on the barrier function, it's possible inhibiting several cytokines at the same time would have stronger effects.

#### Larazotide

Larazotide acetate, also known as AT-1001, is a synthetic peptide derived from the *Vibrio cholerae* zonula occludens toxin (ZO-T or zonulin) which behaves as a zonulin antagonist and proposed as permeability regulator (286) (Figure 3). Zonulin is released by intestinal epithelial cells after diet or microbiota stimuli. Zonulin-mediated detachment of the ZO-1 protein from the TJ protein complex has a direct effect in increasing intestinal permeability (287). Larazotide prevents TJ opening, being able to block zonulin receptors locally, by joining the receptors itself, decreasing TJ detachment and promoting TJ assembly and structural filaments rearrangement (288). Larazotide was developed for the treatment of CD (289) and later tested in type 1 diabetes, inflammatory bowel disease, Kawasaki disease, respiratory diseases (290), collagen-induced arthritis (291) and intestinal ischemic injury (292, 293).

Four clinical trials using larazotide acetate have been published, all in CD (289, 294–296). These studies confirmed its safety and efficacy for reducing gluten-induced symptoms as well as an interesting inverse dose effect, that is, greater reduction of symptoms with lower doses. Nevertheless, positive results cannot be linked to a reduction of small bowel permeability, measured by the lactulose-mannitol test, due to huge variability, leading to controversial results. A phase 3 trial is ongoing (ClinicalTrials.gov Identifier: NCT03569007) to test larazotide in lower doses in CD patients on a gluten-free diet. Hence, although some clinical benefit has been observed, a more accurate evaluation of larazotide effect on intestinal permeability is needed, not only in CD but also for other pathologies with paracellular intestinal barrier dysfunction.

### Lubiprostone

Lubiprostone (LBP) is a prostaglandin E1-derivative able to bind and activate the chloride channel type 2 (ClC-2) located in the luminal side of the epithelium, improving bowel frequency and stool consistency in constipated-IBS patients (297–301) (Figure 3). Moreover, LBP has been reported to enhance intestinal barrier function, reversing IFNγ-induced decrease in TEER and the increase in fluorescein labeled-dextran permeability and enhancing the expression of CLDN-1 *in vitro* (302).

LBP reduced the severity of colitis as well as intestinal permeability in both DSS and TNBS-induced colitis in murine models (303). Alternatively, when LBP was administered to ClC-2 knockout mice, the protective effect against DSS colitis was limited, suggesting a central role of chloride channels in the restoration of barrier function and TJ architecture driven by LBP (303). LBP also reduced mannitol flux in ischemia-injured intestine in *ex vivo* porcine models (304), and decreased chronic water avoidance stress-induced visceral hyperalgesia in rats, partly by down-regulation of OCLN and also up-regulation of CLDN-2 in rat colon crypts (305, 306). The potential of LBP to prevent small intestinal injury and increased permeability related to non-steroidal anti-inflammatory drugs has been reported in a rat model (307).

One study assessed the effect of LBP on human intestinal barrier function after administration of diclofenac, showing a significant reduction of lactulose-mannitol ratio compared to the control group (308). Unfortunately, the three randomized trials that support the use of LBP in IBS with constipation did not evaluate intestinal permeability as an endpoint.

### Dexmedetomidine

Dexmedetomidine (DMM) (Figure 3) is a highly selective 2-adrenoreceptor agonist, used as a sedative and anesthetic adjuvant. Interestingly, it also shows a protector role against barrier dysfunction and intestinal injury. However, the exact mechanisms are not completely elucidated, although it is able to accelerate intestinal wound healing by increasing intestinal epithelial cell proliferation (309). Pretreatment with DMM reduced intestinal injury in a rat model of intestinal ischemia (310), and also improved intestinal microcirculatory dysfunction and barrier dysfunction in endotoxemic rats (311) in association with a reduction of OCLN cleavage and bacterial influx into the spleen. After traumatic brain injury, GI system dysfunction and impairment of barrier function are common features (312). DMM was able to reduce systemic inflammatory cytokines and barrier dysfunction, and to improve villus structure in a rat model of brain injury (313). DMM has also proved to protect against heat stroke-induced inflammatory response and multi-organ dysfunction (314). DMM also demonstrated capacity to reverse burn-induced intestinal epithelial hyperpermeability by reducing inflammation and enhancing the expression and distribution of the TJ proteins ZO-1 and OCLN (315).

In humans, a randomized, double-blinded trial using either perioperative DMM or placebo in patients who underwent an hepatectomy not only showed a decrease in clinical relevant biomarkers of intestinal injury but also a reduction of intestinal failure scores at 72 h after surgery (316). In another randomized, double-blinded prospective study, DMM enhanced the recovery of GI and reduced intestinal injuries and permeability, reflected by decreased serum diamine oxidase and intestinal fatty acid-binding protein expression (317). A recent randomized double-blinded prospective study, suggests DMM as a more suitable anesthetic for patients undergoing GI surgery as it is associated with a decrease in TNF-α, and *D*-Lactate along with an increase in the activation of α7nAChR (318).

### Mast Cell Stabilizers and Flavonoids

Intestinal mast cells (MCs) (Figure 3) play an essential role in barrier function regulation and gut homeostasis as shown both *in vitro*, in animal models and in humans as reviewed elsewhere (216). MC activation leads to the release of a wide variety of proinflammatory and regulatory mediators, and many of them all have an effect on intestinal barrier as well as modulating immune response and enteric nervous system. Though the inhibition of MC activation has been extensively investigated, and many different approaches are possible (319–321), the use of MC stabilizers has gained some consideration in the management of several intestinal disorders in humans, mainly because its beneficial role in the regulation of IEB is based on a solid and vast literature in preclinical models (228, 322–332). Among MC stabilizers, only ketotifen and disodium cromoglycate (DSCG), have been translated to the clinic.

There are few studies exploring the effect of MC stabilizers in modulating IEB in humans. In one study, ketotifen was able to reestablish GI permeability in a small group of food allergy patients (333). In a trial with IBS patients, ketotifen reduced several IBS symptoms, although barrier function was not explored (334). Although preliminary, ketotifen has also shown significant benefits for the treatment of post-operative ileus (335), a condition that seems to be also related with dysfunction of IEB (336). DSCG reverted the increase of intestinal permeability triggered by CRF or stress in healthy volunteers (224). Previously, DSCG pretreatment reduced milk-induced in intestinal permeability, in children with cow's milk allergy (337, 338) or food allergy (339), and in patients with dyshidrotic eczema (340) but nor in atopic eczema (341), although these studies were performed in small groups. Several other studies have shown the potential utility of DSCG for IBS treatment (342–344), but again, little clinical evidence is available to support *its* use as a possible modulator of the IEB.

Flavonoids are natural substances with variable phenolic structures commonly present in fruits, vegetables, tea, wine, grains, bark, roots, stems, and flowers (345). Flavonoids present a natural antioxidant, antimicrobial, cytoprotective, and anti-inflammatory activity (346). Multiple *in vitro* studies show the ability of several flavonoids, including 8-prenylnaringenin, anthocyanins, berberine, puerarin, genistein, kaempferol, naringenin, quercetin, and luteolin, among others, to restore barrier dysfunction, predominantly in a Caco-2 cells (347–354). Moreover, it has been described their ability to increase the expression, assembling and production of different *TJ proteins* such as ZO-1 and 2, OCLN and *CLDN 1, 3*, and 4 through the activation of AMPK and the inhibition of NAPDH oxidase /NF-γB and MLCK and MLC phosphorylation (244, 352, 353, 355–357). *In vivo* studies in rat, highlight the effect of flavonoids in the upregulation of several pathways involved in the expression of several TJ proteins 1 (351, 357, 358) In humans, we are awaiting for ongoing clinical trials to determine the role of natural flavonoids in the management of IEB dysfunction (346).

### Glucocorticoids

Glucocorticoids (GCs) (Figure 3) play an important role in maintaining homeostasis through anti-inflammatory and immunosuppressive actions (359), mediated mostly through GC receptors (360) GCs synthetic derivatives are essential in the clinical treatment of inflammatory and autoimmune diseases (361).

GCs are released after barrier disruption, in part to neutralize the effect of TNF, *via* inhibition of MLCK activity (362). In the same *in vitro* model, GCs triggered a time and dose-dependent increase in TEER in a GC receptor-dependent manner although no changes were observed in TJ architecture (362). GCs also regulated CLDN expression *via* MKP-1 in cell lines (363), but also in human and rat colon mucosal crypts (364). In addition, it has been described that GCs modulate the expression of several other molecules related with TJ polarization and development (365). An interaction between GCs and IL-10 p38 MAPK improved barrier integrity after TNF-α *challenge* in a Caco-2 model (366). GC receptor deficiency aggravated barrier integrity in an animal model of colitis (367).

GCs reduced intestinal permeability in a large number of patients, mostly in Crohn's disease (368, 369), but also in the rectum of collagenous colitis patients (370). In addition, the effect of GCs on permeability is not restricted to IEB because similar modulatory effects have been shown in lung epithelia (371) and the blood brain barrier (372). In addition, the use of GCs for treating intestinal inflammation during sepsis has been proposed to reduce intestinal barrier dysfunction (373). Finally, UC patients display decreased levels of the liver receptor homolog-1 (LRH-1) in the colon (374, 375). LRH-1 is involved in the replacement of the adrenal steroidogenic factor 1 and GC synthesis in the adrenal medulla (376). A recent study has shown how LRH-1 restoration reestablished epithelial integrity in mouse and human organoids as well as its overexpression protected mice from developing colitis (377).

### Mucoprotectors

Mucoprotectants are compounds of different nature (insoluble salts, hemicellulose, tannic acid, gelatins…) with the ability of enhancing the intestinal barrier by creating a film-forming barrier over the intestinal mucosa (Figure 3), helping to reduce the effect of pathogens and to improve the function of the intestinal barrier (378). These compounds work intraluminally to modify enteric contents and may represent an alternative or complementary therapy for dealing with acute and chronic diarrheal disorders (379, 380).

#### Xyloglucan

Xyloglucan (XG) is a water-soluble, high molecular weight branched polysaccharide hemicellulose. XG helps to reduce permeability changes, preserving TJ, and invasion by *E. coli* in Caco2/Goblet cells (381), and binding to MUC1, in mice exposed to DSS (382). XG is non-toxic and resistant to digestive enzymes, reaching the colon unaltered, where it is partially broken down to oligosaccharides by bacterial endo-ß-glucanases, followed by bacterial fermentation of oligosaccharides (383, 384). The molecular structure of XG is known to possess mucomimetic and mucoadhesive properties (382). XG is often combined with gelatin or gelose to prolong its availability within the intestine, but showing similar protective effects as XG alone on barrier function in rats after *Salmonella enterica* and *Enterococcus hirae* infections (385). The combination of XG, pea proteins, tannins from grape xylo-oligosaccharides also offered protection against stress-induced visceral hypersensitivity and intestinal hyperpermeability in rats (380).

In humans, several clinical trials have shown the efficacy of XG in the treatment of acute diarrhea in children (386, 387) and adults (388), and also in chronic diarrhea in IBS patients, improving the majority of symptoms (389, 390). So far, these findings have not been linked to its ability to regulate IEB, and additional trials are needed to support this concept.

#### Gelatine Tannate

Gelatine tannate (GT) is a complex of tannic acid and gelatin which forms electrostatic bonds with mucin to create a protein-based biofilm over the intestinal mucosa (391, 392). Gelatin is a collagen derivate, which is ingested as an insoluble powder at acidic pH, that becomes a gelatin at pH > 5.5 (393). In the intestine, this complex increases the epithelial resistance against *E. coli*, contributing to restore the normal physiology of barrier function in Caco and Goblet cells™ (394). GT also helps to restore the mucus layer and to modulate the intestinal microbiota in the DSS model of murine colitis (395), and in Caco-2 cells, where it prevents the release of TNF-α induced by LPS (396). Furthermore, tannins allow the precipitation of pro-inflammatory molecules from the intestinal mucus and their fecal elimination (396). Together, these observations may explain the protective effect of GT on intestinal barrier function.

Several clinical trials have been performed with GT for acute diarrhea in children, and adults, with mixed results (379, 397, 398). The combination of GT and tyndallized probiotics has been claimed as highly effective in the treatment of moderate and prolonged diarrhea, but clinical evidence awaits the results of an ongoing clinical trial (ISRCTN63068134). Similar to XG, additional evidence is needed to link the positive effects to the protective effect on IEB.

#### Diosmectite

Diosmectite is a medicinal clay (aluminum and magnesium silicate) frequently used as an adjuvant therapy in children and adults with acute diarrhea (399, 400), to reduce stool output, to provide symptomatic relief and to prevent dehydration (398). The mechanism of action is complex, but partly related to modifications of the rheological characteristics of the GI mucus barrier, to reduce penetration of toxins, adsorptive properties, reduction of intestinal permeability by increasing the expression of OCLN, CLDN-1, and ZO-1, and increased MUC2 expression. These mechanisms have been replicated mainly in Caco-2 and HT-29 cell lines, and in rodent and piglets animal models in response to TNF, acetic acid or TNBS (401–405).

Diosmectite has been shown to improve acute and chronic diarrhea, based on a number of open and randomized double-blind, placebo-controlled clinical trials, performed mostly in children with acute diarrhea, and highlighted in a recent Cochrane review (398). Nonetheless, the clinical benefit has not been associated with its barrier protective characteristics.

### Epigenetic and Exosome-Mediated Regulation of Intestinal Barrier Function

In the last decade, exosomes (food and host-derived) and enclosed micro-RNA (miRNA)s' role as modulators of immune responses and IEB function has been widely reported. miRNAs are small (21–23 bp) non-coding RNAs that regulate gene expression either by binding to the 3′ untranslated region of their target mRNAs or *via* endonucleolytic mRNA cleavage, promoting post-transcriptional repression and influencing intestinal homeostasis (406, 407). miRNAs have been implicated in several GI physiologic and pathophysiologic mechanisms and studied widely in intestinal immune and inflammatory diseases, including IBS and IBD, though studies are highly heterogeneous.

Both *in vitro* and *in vivo* assays have recently shown that after IL-1β administration both Caco-2 cells and enterocytes from mice with colitis display increased small intestinal TJ permeability, a rapid increase in miR200C-3p and reduced levels of OCLN mRNA and protein, meanwhile the antagomiR-200c prevented OCLN and permeability changes (408). Moreover, colon tissues and organoids from patients with UC had increased levels of IL-1β mRNA and miR200C-3p compared with healthy controls. In other studies, transfection of miR-21 in Caco-2 cells also resulted in the loss of TJ as well as ultrastructural changes enhancing intestinal permeability through the degradation of RhoB and PTEN (409, 410). An increase of miR-21 and miR-126 has been also observed in colon, feces and blood of UC, and CD patients (409, 411). In addition, increased expression of miR-122a has been also noticed in Caco-2 cells after TNF-α exposure, increasing barrier permeability through the degradation of OCLN mRNA (412).

By inactivating the endonuclease RNase Dicer, enzyme responsible of the pre-miRNAs cleavage and the following maturation to functional miRNAs (413), McKeena et al. showed altered expression of Cadherin 1 and Cathepsin B. Dicer1 mutants also displayed impaired epithelial barrier function, most probably due to the disorganization of the epithelial layer and the junctional complexes (414). Hence, the role of miR-144 in downregulating OCLN and ZO-1 expression and enhancing intestinal permeability has been reported in a rat model od IBS-D (415).

Other miRNAs, such as miR-93 and miR-29a/b seem to offer protective effects on barrier function. miRNA-93 is responsible of PTK6 downregulation in YAMC cells, reversing the effects in permeability caused by TNF-α and IFN-γ (416). miR-29a and miR-29b prevented inflammation in mice after DSS-induced colitis when delivered with supercarbonate apatite nanoparticle (417). Increased levels of miR-29a have also been found in blood, small bowel and colon in IBS and colon in IBD patients (113, 418, 419), but, as a matter of fact, its role is sometimes contradictory (420). miR-29a overexpression increased epithelial permeability by targeting glutamine synthetase gene (GLUL), alteration that has been previously associated with increased membrane permeability (113). Moreover, in IBS-D patients, increased levels of miRNA-29a and miRNA-29b have been described together with a reduction of CLDN1, ZO-1 and nuclear factor-kB repressing factor, while increased levels have also been observed in mice models of IBS or colitis (113, 419).

After showing a correlation between differential mRNA expression and ultrastructural changes in the epithelium of IBS-D patients (421), the same group reported the ability of miR-16 and miR-125b to modulate barrier function in the jejunum of these patients through the regulation of claudin-2 and cingulin expression, respectively (422). In a recent study, Martínez et al. reported several regulated miRNAS in the rectal mucosa of post-infectious-IBS patients along with downregulation of their target mRNAs involved in barrier function (423). Finally, Mahurkar-Joshi et al. (424) have reported decreased levels of miR-219a-5p and miR-338-3p in sigmoid biopsies in IBS, particularly in IBS-C. Inhibition of miR-219a-5p resulted in altered expression of proteasome/barrier function genes and enhanced permeability of intestinal epithelial cells. Additionally, inhibition of miR-338-3p in cells caused alterations in the mitogen-activated protein kinase (MAPK) signaling pathway genes (424).

Not only host miRNA but also plant and bacterial-derived miRNAS have gained special attention for their potential role, yet disputed, as cross-kingdom gene expression regulators, influencing plant interactions with animals and microorganisms to regulate a number of physiological functions (425). In humans, diet would be a primary source of plant miRNA uptake, whereby have been termed as xeno-miRNAs (xenomiRs), moreover, effective detection and quantification of dietetically absorbed plant microRNAs in human plasma (426). Several mechanisms, including transmembrane miRNA carriers, receptor-facilitated endocytosis, phagocytosis, macropinocytosis, clathrin-mediated, caveolin-mediated, and clathrin and caveolin-independent endocytosis, paracellular diffusion, and luminal immune cell capture have been proposed to explain xenomiRs uptake by intestinal epithelial cells, though, to date, still unresolved (425, 427). Later, xenomiRs could be packaged into microvesicles (426) or associated with proteinase K-resistant complexes (428) to be transported and released into the bloodstream to reach out target tissues. It has been shown that absorption of more stable xenomiRs, such as miR-2911, could be promoted when intestinal permeability is enhanced, which may be of use for engineering delivery of dietary miRNA (429). Moreover, gut microbiota could be responsible for enhancing xenomiRs absorption, increasing their bioavailability by degrading the exosomes components (430).

Despite the well-known role of xenomiRs in shaping microbiota and modulating inflammation and immune activation (410, 430, 431), including the down-regulation of TNF receptor (432), direct regulation of the epithelial barrier has not been reported (430). However, given TNF-α ability to modulate barrier permeability, xenomiRs could be indirectly involved in the regulation of IEB. In addition, several studies have shown the ability of plant-derived exosomes, enriched for diverse miRNAs, to contribute to intestinal barrier integrity (410, 433–435), though, again, a direct link could not be established and the effect on barrier function could be dependent on other non-xenomiR-related mechanisms. Because some plant xenomiRs modulate the expression of enterocyte transporters (436), cytokines involved in barrier function such as TNF-α or IL-1β, and activate Wnt/β-catenin pathway (437), it remains plausible that xenomiRs could act as regulators of intestinal barrier permeability. Furthermore, gut microbiota, by enhancing xenomiRs absorption, may also regulate the expression of different miRNAs in IECs (438).

Therefore, considering miRNAs promising role on modulating intestinal barrier function, miRNA based therapeutic strategies are moving from bench to the bedside. Although with several limitations, regulation of miRNAs expression can be achieved by administering synthetic miRNAs or miRNAs expressing vectors or by anti-sense nucleotides (439). Their therapeutic application for barrier function and intestinal inflammation and cancer is under development though further research is needed (440–442).

## Summary and Conclusions

This is an exciting time for intestinal permeability. We have gone from not recognizing the importance of intestinal permeability, no more than 30 years ago, to express now that most digestive and extradigestive disorders have something to do with it. Probably, the truth lies somewhere between these two extremes. What is certain is that pathophysiological, functional and molecular knowledge has advanced enormously in that period of time, and with it, the search for better therapeutic options to manage intestinal permeability disorders. In this review, we have discussed the most relevant therapeutic approaches to improve intestinal permeability and barrier dysfunction. To move forward, more clinical trials, new molecules intended directly to fine tuning of intestinal permeability, differential treatments according to the affected intestinal segment, according to sex, according to age, and to many other variants are needed. In addition, it is important to recognize the role of other factors, such as immune activation and microbiota-related regulation of barrier defects in order to deal better with chronic inflammation in the gastrointestinal tract because presumably targeting only the barrier may not be sufficient to change the natural history of many of these conditions. The advancement in our knowledge of the intimate mechanisms and the inducing and predictive factors of changes in permeability will lead to the development of a better therapeutic approach in several digestive and also extradigestive diseases.

## Author Contributions

MF, MA-B, MA-G, JP-GM, XS-R, AH-P, EE, AG-C, DG, BL, and CA-C reviewed scientific literature and collected data. MF, MA-B, MA-G, JP-GM, XS-R, AH-P, CA-C, and JS wrote the paper. MF, MA-B, and MA-G prepared the tables and figures. All authors critically reviewed and edited the manuscript in its final version and approved the final draft of the manuscript.

## Funding

This work was supported in part by Fondo Europeo de Desarrollo Regional (FEDER), Fondo de Investigación Sanitaria and Centro de Investigación Biomédica en Red de Enfermedades Hepáticas y Digestivas (CIBEREHD), Instituto de Salud Carlos III, Subdirección General de Investigación Sanitaria, Ministerio de Economía y Competitividad, Ajuts per a la contractació de personal investigador FI–Agència de Gestió d'Ajuts Universitaris i de Recerca (AGAUR), Generalitat de Catalunya; The Swedish Research Council, and the European Commision. PI19/0164 (BL), FI20/00256 and 2020FI_B1 00127, 2019FI_B 00817 (MA-B); PI17/0190 (JS); CIBERehd CB06/04/0021 (JS and CA-C); dnr 2019-00653 (JP-GM); GA no:848228 (JS, CA-C, EE, AG-C, and BL). MF received support from FWO (Fonds Wetenschappelijk Onderzoek) grant G.093818 to P. Vanden Berghe, KU Leuven.

## Conflict of Interest

CA-C discloses past scientific collaboration with Noventure S.L. JS has served as consultant for Noventure and discloses present and past recent scientific collaborations with Salvat, Norgine, Alfa-Sigma, Cosmo, Adare, Devintecpharma, Pileje and Danone that do not constitute a conflict of interest in developing the content of the present manuscript. The remaining authors declare that the research was conducted in the absence of any commercial or financial relationships that could be construed as a potential conflict of interest.

## Publisher's Note

All claims expressed in this article are solely those of the authors and do not necessarily represent those of their affiliated organizations, or those of the publisher, the editors and the reviewers. Any product that may be evaluated in this article, or claim that may be made by its manufacturer, is not guaranteed or endorsed by the publisher.

## Abbreviations

AMPK: AMP-activated protein kinase
Arg: Arginine
CD: Celiac Disease
ClC-2: Chloride channel type 2
CLDN: Claudin
CRF: Corticotropin-releasing factor
CRF-R: CRF Receptor
DF: Dietary Fibers
DMM: Dexmedetomidine
DSCG: Disodium cromoglycate
DSS: Dextran sodium sulfate
EcN: *Escherichia coli* Nissle 1917
EV: extracellular vesicles
FODMAP: Fermentable oligosaccharides, disaccharides, monosaccharides, and polyols
GC: Glucocorticoids
GI: Gastrointestinal
Gln: Glutamine
GLP-2: Glucagon-like peptide 2
GLP-2R: Glucagon-like peptide 2 receptor
GOS: Galactooligosaccharide
GT: Gelatine tannate
IBD: Inflammatory Bowel Disease
IBS: Irritable Bowel Syndrome
IBS-D: Irritable Bowel Syndrome with Diarrea
IE-IFG-1R: Epithelial insulin-like growth factor-1 receptor
IEB: Intestinal epithelial barrier
IFN-γ: Interferon gamma
Ig: Immunoglobulin
IL: Interleukin
LBP: Lubiprostone
LPS: Lipopolysaccharide
LRH-1: Liver receptor homolog-1
MC: Mast cell
MiRNA: Micro-RNA
MLC: Myosin light chain
MLCK: Myosin light chain kinase
NF-KB: Nuclear factor kappa B
NLRP3: NLR family pyrin domain containing 3
OCLN: Occludin
SCFA: Short-chain fatty acids
TEER: Transepithelial electrical resistance
TJ: Tight Junction
TLR: Toll-Like Receptor
TNF-α: Tumor necrosis factor
UC: Ulcerative colitis
VDR: Vitamin D receptor
Vit: Vitamin
XenomiRs: xeno-miRNAs
XG: Xyloglucan
Zn: Zinc
ZO: *Zonula Occludens*.
